# A simple and accessible CRISPR genome editing laboratory exercise using yeast

**DOI:** 10.17912/micropub.biology.000699

**Published:** 2023-02-06

**Authors:** Connor Shortt, Elise Krippaehne, Brian M Wasko

**Affiliations:** 1 College of Osteopathic Medicine of the Pacific Northwest, Western University of Health Sciences, Lebanon, Oregon, USA

## Abstract

CRISPR is a revolutionary tool to engineer the genome. Herein, we describe a laboratory exercise designed to introduce students to CRISPR by editing the genome of yeast to disrupt the *ADE2* gene, which results in yeast that form red colored colonies. The experiment was constructed to be performed in a single laboratory session and to be accessible to students and instructors without experience working with yeast.

**Figure 1.**
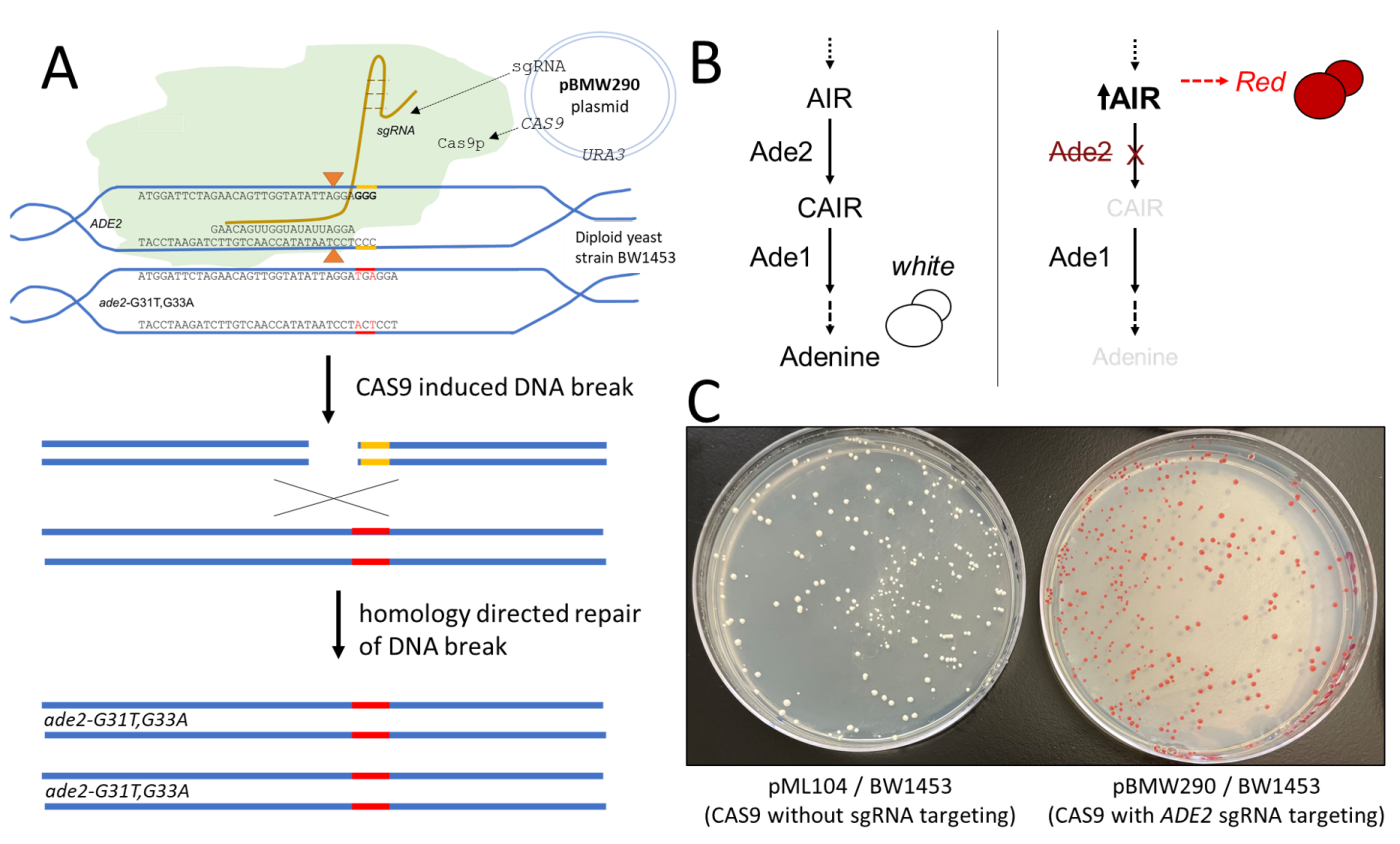
**(A)** Diagram of CRISPR/CAS9 targeting of the *ADE2* gene using pBMW290 plasmid and BW1453 diploid yeast strain containing a wildtype *ADE2* copy and a mutant *ade2* allele containing a premature stop codon. **(B)** The purine biosynthetic pathway in the presence of Ade2 protein results in yeast that display white colonies, while cells lacking Ade2 activity form red colored colonies due to the accumulation of a pigment in the vacuole. AIR is 5-aminoimidazole ribonucleotide and CAIR is 4-carboxyaminoimidazole ribonucleotide. **(C)** Experimental results of BW1453 diploid yeast transformed with pML104 (CAS9 only, no sgRNA targeting) or pBMW290 plasmid (CAS9 and sgRNA targeting *ADE2* ) and grown on media lacking uracil for plasmid selection.

## Description

Clustered regularly interspaced short palindromic repeats (CRISPR) in conjunction with a CRISPR associated protein such as Cas9 from *Streptococcus pyogenes* , has been leveraged into a precise and accessible genome editing tool. The potential uses of CRISPR as a treatment for a wide array of medical conditions has made teaching its applications to undergraduate science students increasingly pertinent.

CRISPR/Cas9 (for a detailed review, see Thurtle-Schmidt and Lo 2018), has been engineered to require two parts: the Cas9 nuclease protein that cleaves double-stranded DNA (dsDNA), and a single guide RNA (sgRNA) that contains scaffolding to bind to Cas9 and a targeting region of ~20 nucleotides that can be engineered to be complementary to a site in the genome which also contains a protospacer adjacent motif (PAM) sequence (Figure 1A). A PAM site (e.g., NGG for Cas9, where N is any nucleotide and G is guanine) is required for Cas9 to bind the target sequence. CRISPR/Cas9 creates dsDNA breaks upstream of the PAM site, which induces cell cycle arrest in cells that contain a sgRNA targeted sequence. DNA double-stranded breaks such as those induced by Cas9 can be repaired by the homology directed repair (HDR) pathway or non-homologous end joining pathway. If a Cas9 induced DNA break is repaired with high fidelity, the continued presence of CRISPR/Cas9 would recleave the DNA. The HDR repair pathway can be leveraged by having a dsDNA repair template (commonly exogenously added DNA) that is homologous to the CRISPR targeted DNA region, except with alterations of the PAM site (i.e., either or both of the GG nucleotides changed to A, C, or T) which allows for cells that incorporate the changes present in the repair template to no longer undergo a Cas9-induced dsDNA break and proceed through the cell cycle.

We set out to develop a robust laboratory exercise that would allow use of CRISPR in *Saccharomyces cerevisiae* (yeast) in teaching labs using a single session of instruction. Protocols and suggested reagents are included in a manner meant to assist instructors without previous yeast research experience. A plasmid (pBMW290) was generated with a sgRNA that targets near the beginning of the *ADE2* gene. *ADE2* encodes an enzyme involved in purine biosynthesis and was selected because loss of Ade2 protein results in the build up of a pigment in the vacuole that causes the formation of red colonies (Figure 1B). A diploid strain was constructed that is heterozygous at the *ADE2* allele with one wildtype copy and the other copy containing mutations (G31T and G33A) that result in a premature stop codon and alteration of the PAM site required by the pBMW290 sgRNA. This strain (BW1453) when transformed with the pML104 plasmid that does not encode an sgRNA targeting region remains white, as Cas9 is not targeted anywhere in the genome to digest DNA (Figure 1C). When pBMW290 is transformed into BW1453, the wildtype *ADE2* gene is targeted, which would result in a DNA damage induced cell cycle arrest by itself. However, if the wildtype *ADE2* allele with the double stranded break is repaired using homology directed repair with the mutant *ade2* allele, then this can result in two *ade2* alleles that lack PAM sites and are therefore refractory to the sgRNA targeting and are able to grow, but also lack any Ade2 protein activity and grow as red colonies (Figure 1C). In addition to homology directed repair, it is also possible that the CRISPR-induced dsDNA break could be repaired by the non-homologous end joining (NHEJ) in a manner that results in loss of the functional allele and therefore is another possible mechanism that could yield red colonies. The diploid yeast strain in this study was specifically constructed to bypass the need for an exogenous repair template in order to make the system not require preparation (e.g., via PCR) or purchase of synthetic oligonucleotides which results in cost and time savings. This experimental design is optimized to be performed in a single laboratory session, and the experimental protocol (included below) is meant to be robust and simple for students, and easily accessible for instructors.

## Methods

*Plasmid cloning:*

pML104 was a gift from John Wyrick (Addgene plasmid # 67638 ; http://n2t.net/addgene:67638 ; RRID:Addgene_67638). Oligonucleotides for cloning were oBW784 (GATCGAACAGTTGGTATATTAGGAGTTTTAGAGCTAG) and oBW785 (CTAGCTCTAAAACTCCTAATATACCAACTGTTC) which were first hybridized to make double stranded DNA by mixing equal molar amounts and heating in a thermocycler to 95C for 10 minutes and then allowing to cool to room temperature. dsDNA was then cloned into SmiI and BglI digested pML104 using T4 DNA ligase (New England Biolabs) and transformed into competent TOP10 E.coli (Invitrogen). Cloning was verified by colony PCR using oBW785 and M13R (TTTCACACAGGAAACAGCTATGAC) using APEX Taq Red Master Mix (Genesee Scientific, San Diego, CA) with thermal cycler conditions of: 95 °C 5 min, 32 cycles of 95 °C 30 s, 55 °C 30 s, 72 °C 60 s, and then 72 °C for 5 min, and the presence of an ~400bp PCR product was used to confirm the presence of a desired insert. The resulting plasmid was named pBMW290 and the plasmid was also sequenced (Plasmidsaurus, Eugene, OR), and the sequenced sample was annotated to have undergone dimerization.

*Yeast strain generation:*

Plasmid pBMW290 was transformed into wildtype yeast strain BY4742 (S288C derived, Brachmann et al. 1998) using the Frozen EZ yeast transformation II kit (Zymo Research, Irvine, CA) with and without hybridized repair template consisting of oBW786 (atggattctagaacagttggtatattaggaTgAggacaattgggacgtatgattgttgaggcag) and oBW787 (ctgcctcaacaatcatacgtcccaattgtccTcAtcctaatataccaactgttctagaatccat) and plated onto SD-URA agar. From a plate with repair template oligonucleotides present, a single red colony was selected (BW1451 *ade2* -G31T,G33A) and mated to wildtype BY4741 to generate a heterozygous diploid strain that was named BW1453 ( *ADE2/ade2* -G31T,G33A). Both BW1451 and BW1453 strains were sequence verified to contain the indicated *ADE2* allele(s).

***Laboratory Exercise Protocol***

*Pre-lab preparation:*

*Plasmid preparation*

Sufficient plasmid pBMW290 and pML104 should be prepped using a plasmid prep kit or other desired method. Multiple mini-preps could be pooled together or a midi or maxi type prep could be performed to get a large amount of plasmid DNA so this step is infrequently needed. Grow the *E.coli* w/ pML104 or pBMW290 plasmid in LB + 100 µg/ml Ampicillin media to maintain plasmid selection.

*Storage*

The plasmid DNA can be stored at -20 °C indefinitely and E.coli containing the plasmids can be put into 20% glycerol and stored at -80 °C indefinitely. For use, a small amount of frozen material can be scraped from the frozen tube stock using a sterile pipette tip and transferred to liquid LB+Amp which can be grown overnight with shaking at 37 °C and then used for plasmid preparation.

Long term storage of yeast strains can be performed by mixing 750 µl of yeast grown in a YPD overnight into 750 µl of sterile 50% glycerol in a cryovial tube for storage indefinitely at -80C. Upon desired use, a small amount of frozen material can be scraped from the frozen tube stock and grown at 30 °C for 2-4 days on YPD agar plates, which can then be stored at 4 °C.

*Yeast SD-URA agar plate preparation*

Add 47.47 g of SDA-Ura powder per 1 liter of water and mix. Autoclave at 121 °C, 15 PSI for 20 minutes. Let cool enough to be able to safely handle, then pour ~25ml per 100 mm petri dish. Let plates dry overnight at room temperature and then store plates in a sealed container at 4 °C until use.

*Prelab setup*

The day prior to the lab, BW1453 yeast should be used to inoculate 1.5ml of liquid YPD growth media per each transformation (2 transformations per student/group suggested). Ideally, yeast should be grown with vigorous shaking at 30 °C in a flask overnight, however yeast will also grow at room temperature without shaking if necessary.

*Student Laboratory Protocol:*

## Reagents

Table of yeast strains and plasmids.

**Table:** 

**Yeast strain**	**Strain background**	**Genotype**	**Reference**
BW1451	BY4742	*MATα* , *ade2* - *G31T* - *G33A* , *ura3Δ0* , *his3Δ1* , *leu2Δ0* , *lys2Δ0*	This study
BW1453	BY4743	*ADE2* / *ade2* - *G31T* - *G33A* , *ura3Δ0* / *ura3Δ0* , *his3Δ1* / *his3Δ1 leu2Δ0* / *leu2Δ0 LYS2* / *lys2Δ0 MET15* / *met15Δ0*	This study, mated BW1451 with BY4741

**Table:** 

**Plasmid**	**Features**	**Reference**
pML104	Amp ^R^ , *URA3* , 2µ ORI, *CAS9* , sgRNA scaffold without a targeting sequence	Laughery et al. 2015.
pBMW290	pML104 + sgRNA targeting *ADE2 at* GAACAGTTGGTATATTAGGAGGG	This study

Table of suggested reagents and potential source

**Table:** 

**Suggested Reagents**	**Source (item number)**	
Frozen EZ Yeast Transformation II kit	Zymo Research (T2001)	
YPD growth media	Sunrise Science (1875-250)	
SDA-URA	Sunrise Science (1704-500)	

This protocol was optimized for simplicity and deviations for increased cost efficiency are possible. In place of the transformation kit, other kits could be obtained (e.g., MP Biomedicals EZ transformation kit), or yeast transformation methods can be used by the preparation of necessary reagents (e.g., PEG, lithium acetate, etc. Geitz and Woods 2001). SDA-URA media could also be made using individually purchased reagents (e.g., CSM-URA powder, agar, glucose, yeast nitrogen base, and ammonium sulfate). Note that the amount of adenine present in the plates may be important for the formation of the red color. Yeast extract, bacto peptone, and glucose could be purchased separately and used at 1%, 2%, and 2% w/v final concentrations respectively to make YPD. If no autoclave is available, an instant-pot type pressure cooker could be used (Swenson et al. 2018), or premade plates can be purchased from companies such as Teknova (e.g., product C3080). Mini-microcentrifuges can be sufficient for pelleting yeast, or if necessary, the yeast suspension can settle with gravity and time.

Yeast strains and plasmids are available upon request.

## References

[R1] Brachmann CB, Davies A, Cost GJ, Caputo E, Li J, Hieter P, Boeke JD (1998). Designer deletion strains derived from Saccharomyces cerevisiae S288C: a useful set of strains and plasmids for PCR-mediated gene disruption and other applications.. Yeast.

[R2] Gietz RD, Woods RA (2001). Genetic transformation of yeast.. Biotechniques.

[R3] Laughery MF, Hunter T, Brown A, Hoopes J, Ostbye T, Shumaker T, Wyrick JJ (2015). New vectors for simple and streamlined CRISPR-Cas9 genome editing in Saccharomyces cerevisiae.. Yeast.

[R4] Swenson VA, Stacy AD, Gaylor MO, Ushijima B, Philmus B, Cozy LM, Videau NM, Videau P (2018). Assessment and verification of commercially available pressure cookers for laboratory sterilization.. PLoS One.

[R5] Thurtle-Schmidt DM, Lo TW (2018). Molecular biology at the cutting edge: A review on CRISPR/CAS9 gene editing for undergraduates.. Biochem Mol Biol Educ.

